# Breakthrough to Non-Vacuum Deposition of Single-Crystal, Ultra-Thin, Homogeneous Nanoparticle Layers: A Better Alternative to Chemical Bath Deposition and Atomic Layer Deposition

**DOI:** 10.3390/nano7040078

**Published:** 2017-04-06

**Authors:** Yu-Kuang Liao, Yung-Tsung Liu, Dan-Hua Hsieh, Tien-Lin Shen, Ming-Yang Hsieh, An-Jye Tzou, Shih-Chen Chen, Yu-Lin Tsai, Wei-Sheng Lin, Sheng-Wen Chan, Yen-Ping Shen, Shun-Jen Cheng, Chyong-Hua Chen, Kaung-Hsiung Wu, Hao-Ming Chen, Shou-Yi Kuo, Martin D. B. Charlton, Tung-Po Hsieh, Hao-Chung Kuo

**Affiliations:** 1 Green Energy & Environment Research Laboratories, Industrial Technology Research Institute, No. 195, Sec. 4, Chung Hsing Road, Chutung, Hsinchu 31040, Taiwan; alexliao.ep99g@nctu.edu.tw (Y.-K.L.); yungtsungliu@itri.org.tw (Y.-T.L.); itriA00515@itri.org.tw (W.-S.L.); fallout34@itri.org.tw (S.-W.C.); 2 Department of Electro-Physics and Department of Photonic & Institute of Electro-Optical Engineering, National Chiao Tung University, No. 1001, University Road, Hsinchu 30010, Taiwan; khlhjh34@hotmail.com (D.-H.H.); elisa_tien@hotmail.com (T.-L.S.); jerrytzou.ep00g@gmail.com (A.-J.T.); cscbobo@gmail.com (S.-C.C.); eclipse947346@hotmail.com (Y.-L.T.); sjcheng@mail.nctu.edu.tw (S.-J.C.); chyong@mail.nctu.edu.tw (C.-H.C.); khwu@cc.nctu.edu.tw (K.-H.W.); 3 Department of Electronic Engineering, Chang-Gung University, No. 259, Wen-Hwa 1st Road, Kwei-Shan, Taoyuang 33302, Taiwan; sirius0816@hotmail.com (M.-Y.H.); sykuo@mail.cgu.edu.tw (S.-Y.K.); 4 Department of Chemistry, National Taiwan University, No.1, Sec. 4, Roosevelt Road, Taipei 10617, Taiwan; B99203009@ntu.edu.tw (Y.-P.S.); haomingchen@ntu.edu.tw (H.-M.C.); 5 Department of Nuclear Medicine, Chang Gung Memorial Hospital, 5, Fuxing Street, Kwei-Shan, Taoyuang 33302, Taiwan; 6 School of Electronics and Computer Science, University of Southampton, Southampton SO17 1BJ, UK

**Keywords:** nanoparticles, thin-film deposition, chemical bath deposition, thermolysis, atomic layer deposition

## Abstract

Most thin-film techniques require a multiple vacuum process, and cannot produce high-coverage continuous thin films with the thickness of a few nanometers on rough surfaces. We present a new ”paradigm shift” non-vacuum process to deposit high-quality, ultra-thin, single-crystal layers of coalesced sulfide nanoparticles (NPs) with controllable thickness down to a few nanometers, based on thermal decomposition. This provides high-coverage, homogeneous thickness, and large-area deposition over a rough surface, with little material loss or liquid chemical waste, and deposition rates of 10 nm/min. This technique can potentially replace conventional thin-film deposition methods, such as atomic layer deposition (ALD) and chemical bath deposition (CBD) as used by the Cu(In,Ga)Se_2_ (CIGS) thin-film solar cell industry for decades. We demonstrate 32% improvement of CIGS thin-film solar cell efficiency in comparison to reference devices prepared by conventional CBD deposition method by depositing the ZnS NPs buffer layer using the new process. The new ZnS NPs layer allows reduction of an intrinsic ZnO layer, which can lead to severe shunt leakage in case of a CBD buffer layer. This leads to a 65% relative efficiency increase.

## 1. Introduction

Fast, cost-efficient deposition of ultra-thin, high-quality crystalline films on rough or textured surfaces [1] over a large area is needed for semiconductor manufacture. Atomic layer deposition (ALD) has been regarded as inherently useful for thin-film deposition with such strict criteria compared to alternative methods such as chemical vapor deposition (CVD) and physical vapor deposition (PVD) because ALD process allows layer thickness control to nanometer scale [2]. ALD involves a complex multi-cycle chemical process based on self-saturating vapor-phase vacuum deposition. A cycle starts with precursor chemisorption onto the substrate surface, followed by formation of a thin film as reactant is injected [2]. Applications for ALD span metal–oxide–semiconductor field-effect transistor (MOSFET), dynamic random access memory (DRAM), biosensors, waveguide, LEDs and solar cells [2,3]. However, the gaseous-phase growth nature of ALD can limit the crystallinity of the thin film, unless a certain thickness of thin film or certain growth temperature has been achieved [4]. Usually, amorphous-phase material can be partially observed in an ALD-prepared thin film, and can deteriorate device operation, for example causing leakage current in MOSFET [5].

On the other hand, due to the self-limiting nature of ALD, one cycle of ALD deposition only delivers a thin-film layer with a thickness of a few angstroms; therefore, ALD-prepared thin film with over 100 nm thickness could take hours to obtain [3], and its deposition rate depends strongly on the gas source used, which is very expensive and leads to the trade-off between cost and quality source. Also, the ALD-grown thin film can be thermally decomposed or desorbed as growth temperature falls out of the ALD temperature window, leading to requirement of careful control over growth temperature [6]. In terms of hardware setup, many ALD machine setup parameters must be carefully considered to avoid problems, including early condensation of gas source during injection to the chamber, inappropriate design of gas shower head that deters large-area deposition in short growth time, inadequate speed of vacuum pumps extends the time for each ALD deposition cycle. Moreover, due to the nature of ALD, a large amount of precursor/reactor gas has to be pumped and wasted. The need for toxic gaseous waste disposal also goes against cost-efficiency and environmental protection. Overall, the ALD vacuum process is not preferable for deployment to many practical applications on an industrial scale due to its significant cost, complicated procedure and low throughput.

In this paper, we provide a radical new method to form a thin layer of dense sulfide single-crystal nanoparticles such as ZnS, InS, and MoS_2_. The single-crystal nanoparticle layers are synthesized by chemical thermolysis using non-vacuum thermal decomposition by heating a single-source molecular precursor solution drop cast on a substrate. During the synthesis, coalescence of single-crystal nanoparticles forms an ultra-thin, large-area, highly conformal, single-crystal film. This process has the advantages of little liquid waste, little material loss, rapid deposition, and precise control of thickness down to a few nanometers. The new deposition method is also capable of producing thin films with thickness up to 100 nm, and can be thicker if a cyclic process is employed. We propose that this process is competitive to and can potentially replace current established thin-film deposition techniques such as ALD and CBD. Additionally, low temperature fabrication process is becoming more important, due to the fact that the current complementary Metal-Oxide-Semiconductor (CMOS) device requires advanced solution-based metal layer fabrications in back-end packaging. Such thermal decomposition fabrication method is particularly suitable to be integrated into the packaging process.

In order to show the validity of the process in a real device and the “hot” technology sector, we demonstrate the incorporation of a high-quality, nm thick ZnS nanoparticle layer (prepared by thermal decomposition) within a Cu(In,Ga)Se_2_ (CIGS) Photovoltaic (PV) device. We demonstrate that this not only alleviates these issues, but greatly improves efficiency.

Cu(In,Ga)Se_2_ (CIGS) is known to provide the highest efficiency material among thin-film solar cells [7]. CIGS surface typically has roughness of 10–100 nm scale, which presents challenges for deposition of the buffer layer. ALD and chemical bath deposition (CBD) both provide conformal ultra-thin film growth with consistent thickness on nano-patterned substrates, and are particularly suitable for the CIGS surface. Hence, both processes have been utilized to deposit the buffer layer for CIGS solar cells [8]. CBD additionally takes advantage over ALD by its non-vacuum, large-area deposition capability, which makes CBD preferable for the industry. Hence, CBD has become regarded as the most robust industrial deposition method for CIGS buffer layer for decades [9,10,11]. Nonetheless, CBD suffers from issues such as poor crystallinity, high concentration of impurities, and requirements for post process, waste liquid treatment. These issues raise the cost of CIGS solar cells and jeopardize the environment. In our CIGS module fabrication line, a 1 m^2^ CIGS module, the deposition of buffer layer using CBD produces around 10 L toxic chemical waste containing hydrogen sulfide acid, zinc chloride, ammonia [NH_3_] and thiourea [CH_4_N_2_S], while the same film prepared by thermal decomposition only produces less than 10 mL of non-toxic volatile chemicals.

We show that by using a thermally decomposed ZnS nanoparticle layer as replacement for the conventional CBD buffer layer, efficiency of a CIGS solar cell can be boosted by 32%, and issues associated with the CBD process and raising the cost of CIGS solar cells are eliminated. The new ZnS NPs layer allows reduction of an intrinsic ZnO layer, which can lead to severe shunt leakage in case of CBD buffer layer. This leads to a 65% relative efficiency increase.

## 2. Results

The formation of thermally decomposed sulfide nanoparticles starts with a volatile solution with precursor molecule metal diethyldithiocarbamate {[(C_2_H_5_)_2_NCS_2_]_2_X, X = Zn, Cd, Mo, etc.} dispersed in trioctylphosphine (TOP) [P(C_8_H_17_)_3_] dropped on a substrate. The dispersed precursor molecules are then thermally decomposed into sulfide molecules and volatile substances through annealing at 200 °C on a hot plate. The volatile molecules quickly evaporate while sulfide molecules on the substrate become segregated into metal sulfide nanoparticles and coalesced into thin film.

**Figure 1:**
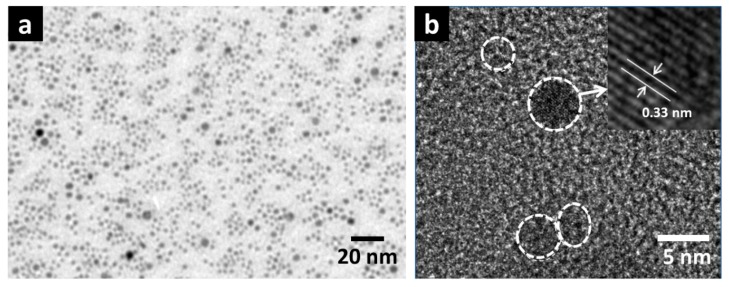
(**a**) TEM image of a small magnification view over ZnS NPs (nanoparticles) grown on copper mesh, and (**b**) a magnification at a few individual ZnS NPs.

ZnS nanoparticles (ZnS NPs) have been chosen in this demonstration for large-area single-crystal nanoparticle layer deposition. Firstly, transmission electron microscopy (TEM) was used to investigate size distribution and crystallinity of the ZnS NPs. For clear TEM observation, ZnS NPs were directly prepared on a copper mesh. The results are shown in Figure 1a,b. In Figure 1a, a view over a broad range of ZnS NPs layer is shown, in which (ignoring a few larger ZnS NPs clusters) the diameter of ZnS NPs is generally consistent (3–5 nm) and homogeneously distributed. A high-resolution TEM (HRTEM) image magnifying one single ZnS NP is shown in Figure 1b, in which individual ZnS NPs are clearly observed and labeled by the white dashed circle. In particular, ZnS NPs show evident crystallinity. Inset to Figure 1b shows a HRTEM image of one single ZnS NPs, in which lattice fringe pattern with an internal spacing of 0.33 nm corresponding to (100) plane of ZnS can be indexed [12]. These TEM images exhibit the high-level crystallinity and homogeneous distribution of the ZnS NPs, while sol-gel grown nanocrystal quantum dots prepared on TEM metal mesh usually show multiple discrete areas of self-assembled quantum dots [13].

The ZnS NPs layer is a perfect alternative to the conventional CBD n-type buffer layer for CIGS solar cells. Usually, CdS or ZnS are chosen to be the n-type buffer layer material, among which, ZnS is more preferable due to its non-toxicity, and better photo-current conversion efficiency between 440 nm and 550 nm visible wavelength range, compared to a CIGS PV device with a CdS buffer layer [14]. However, CBD-prepared thin films are usually amorphous or multi-crystalline and contain complex mixtures. For instance, ZnS prepared by CBD is a mixture of complexes including Zn–S, Zn–OH, and Zn–O [14]. These complexes, such as Zn(OH)_2_, can affect optical and electrical properties of the CBD ZnS film including resistivity and bandgap [15], and operation stability of a CIGS solar cell [16]. The temperature of the most commonly used CBD process is generally between 65 °C and 75 °C, and so subsequent high-temperature process steps, such as deposition and annealing of the transparent conductive oxide (TCO) window layer, can severely deteriorate CIGS devices [17]. This limits the quality of the TCO layer for CIGS solar cells. The growth rate of buffer layer on CIGS through CBD is also differentiated by different crystal orientation of CIGS. Due to the difference in surface energy, growth rates of CBD ZnS on (100)-oriented and (001)-oriented CIGS surface are more severe, while growth rate of CBD ZnS on (221)-oriented CIGS surface is low, causing problems with coverage [18]. Moreover, the CBD has issues of waste liquid, for which expensive and complex post-treatment is needed, and may also increase concern for environmental pollution and ecological impact.

Regarding the above aspects, the ZnS NPs layer prepared by thermal decomposition provides pure single-crystal layers, and has advantage of no material loss, low chemical waste liquid, and simple process, which make ZnS NPs industrially valuable. Since ZnS NPs are prepared under high temperature (below 300 °C in this case), the CIGS PV device with the ZnS NPs layer can be expected to have good heat tolerance. Moreover, the deposition time of thermally decomposed ZnS NPs layers takes only 5 min, while deposition time for CBD ZnS can take 0.5–1 h in order to avoid large-size particle aggregation [19].

**Figure 2:**
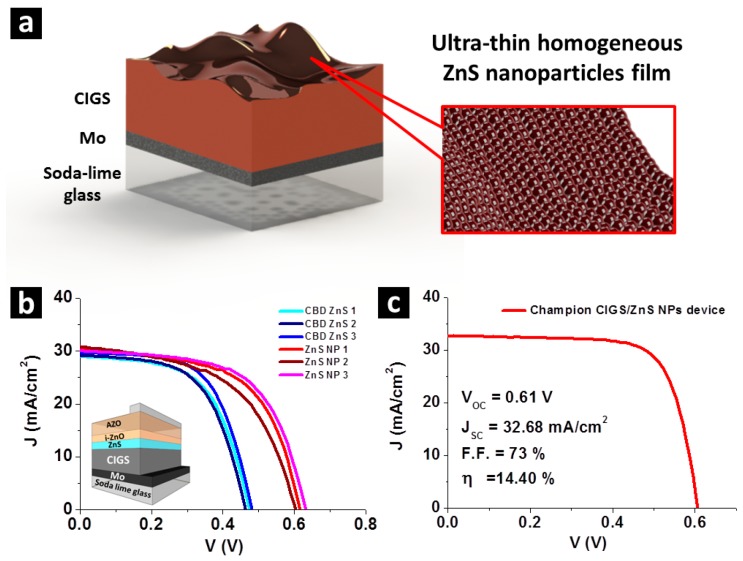
(**a**) A schematic illustration on compact layer of ZnS nanoparticles layer on Cu(In,Ga)Se_2_ (CIGS) surface. (**b**) Current–voltage (J–V) characteristic curves of CIGS solar cells with chemical bath deposition (CBD) ZnS buffer layer and ZnS NPs buffer layer. J–V curves of ZnS NPs devices are presented in pink, red and wine red, while curves obtained from CBD ZnS devices are presented in blue, light blue and navy blue. Absorber layers of the two kinds of CIGS solar cells were separately prepared by co-evaporation. Inset shows a schematic illustration on the device structure. (**c**) J–V curve of Champaign CIGS solar cell with ZnS NPs buffer layer.

CIGS solar cells with the ZnS NPs layer are both prepared for device characterization. The CIGS solar cell with ZnS NPs follows the conventional CIGS solar cell structure, except for the replacement of CBD buffer layer by using ZnS NPs. The device fabrication starts at a rigid glass substrate coated by the 800 nm thick Mo back contact layer, followed by a 2 μm CIGS absorber layer, then, as schematically shown in Figure 2a, an ultra-thin layer of compact ZnS NPs has been conformally coated onto CIGS surface as the buffer layer. The ZnS NPs layer was capped by the 50 nm intrinsic ZnO and the 250 nm Al:ZnO (AZO) window layer. Since ZnS NPs can endure high temperature without any damage, the AZO layer of ZnS NPs devices was prepared at 150 °C (see Methods section), while the AZO layer of the CBD ZnS device is inevitably prepared at room temperature in order to avoid damage to the CBD ZnS layer. The devices were accomplished by deposition of Ni/Al grids as a front electrode. A schematic illustration of a CIGS solar cell has been inset to Figure 2b showing the device structure. Conventional CIGS solar cells with the CBD ZnS buffer layer have also been prepared for comparison. The ZnS/CIGS pn junctions of the two devices are as-prepared for cross-sectional TEM observation. The thickness of ZnS NPs and CBD ZnS has been optimized to reach the highest device efficiency, and will be shown later.

For both CIGS solar cells with the CBD ZnS buffer layer (CBD ZnS device) and ZnS NPs buffer layer (ZnS NPs device), three devices of each type of CIGS solar cell were prepared for quantitative analysis. Current–voltage (J–V) and photon conversion efficiency (PCE) measurements were conducted. Results of J–V curves are shown in Figure 2b, and PCE spectra are shown in Figure S1 in Supplementary Information. In both J–V and PCE diagrams, curves obtained from the ZnS NPs device are presented in pink, red and wine red, while curves obtained from the CBD ZnS device are presented in blue, light blue and navy blue. The CIGS absorber layer of CBD ZnS devices and ZnS NPs devices were separately prepared by co-evaporation. In Figure 2b, where J–V curves are shown, compared to CBD ZnS devices, the ZnS NPs device shows slightly higher short-circuit current (J_SC_) (about 29 mA/cm^2^ for CBD ZnS devices, and about 30 mA/cm^2^ for ZnS NPs devices), and higher open-circuit voltage (V_OC_) (about 0.46 V for CBD ZnS devices, and about 0.62 V for ZnS NPs devices).

J–V characteristic parameters including V_OC_, J_SC_, Fill Factor (FF), and efficiency of all PV devices are collected and shown in Table 1. Comparison has been made to the best efficiency PV devices separately selected from the two families of CBD ZnS devices and the thermally decomposed ZnS NPs device. Best thermally decomposed ZnS NPs devices exhibit comparable J_SC_ to that of the best CBD ZnS device, yet V_OC_ significantly surpasses the best CBD ZnS device. This results in the higher device efficiency of 11.44% for the thermally decomposed ZnS NPs device, in comparison to 8.69% efficiency for the best CBD ZnS device, which has reached 32% relative improvement in efficiency.

With subsequent optimization of the ZnS NPs buffer layer for the CIGS solar cells, we have reduced the thickness of i-ZnO on the ZnS NPs buffer layer from 50 nm to 15 nm. Conventionally, the i-ZnO layer is intended to avoid shunt leakage current caused by bad coverage of the CBD-prepared buffer layer, and the impurities it contains. The quality ZnS NPs layer allows reduction of i-ZnO thickness and acquires more efficient photo-current harvesting. The optimization has increased efficiency to 14.40% (65% relative improvement in efficiency over the conventional cell). The J–V curve of the best device is shown in Figure 2c. V_OC_, J_SC_ and F.F. of the best device are 0.61 V, 32.68 mA/cm^2^, and 73%, respectively. Overall, this result demonstrates viability for the thermally decomposed ZnS NPs layer to replace the conventional CBD ZnS layer.

The enhancement of J_SC_, V_OC_ and efficiency on ZnS NP devices can only be achieved if the quality of the ZnS NC layer is better than that of the CBD ZnS layer in terms of crystallinity, defect concentration and coverage. The thickness of the ZnS buffer layer can also affect device performance because of the high resistance of ZnS and potential barrier which forms at the CIGS/ZnS interface. Investigations into these have been made as follows.

The substrate onto which the thermally decomposed ZnS NPs layer is deposited is a thin film CIGS layer grown on a Mo-coated glass substrate. With the multi-crystalline nature of CIGS, the surface of CIGS inevitably has voids at grain boundaries and roughness of tens of nanometers. For a quality p-n junction for highly efficient CIGS solar cells, it is crucial for the thermally decomposed ZnS NP layer to have a high level of coverage over the CIGS surface in order to create effective depletion region, eliminate shunt leakage, and to protect CIGS surface against plasmon damage during deposition of the above window layer.

**Figure 3:**
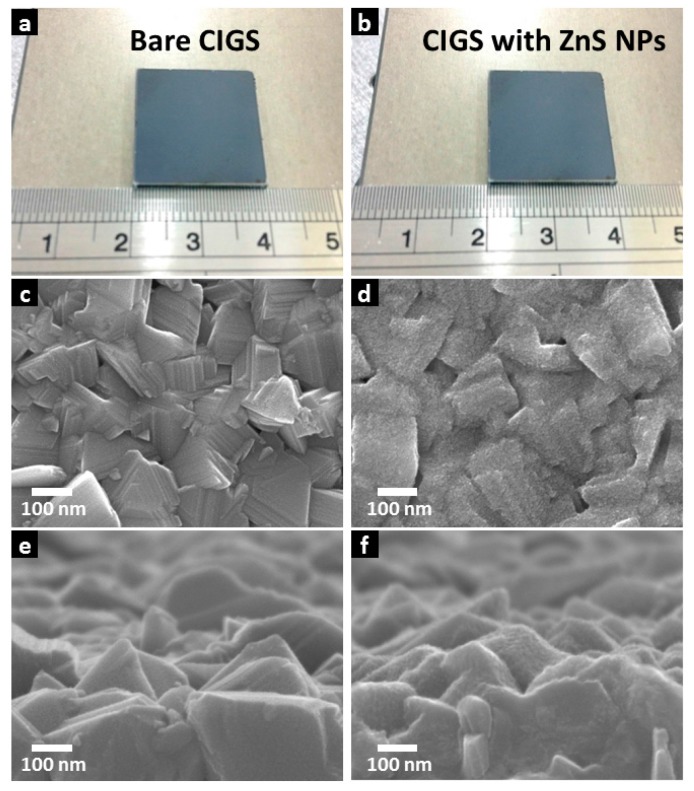
Photograph of a CIGS thin film (**a**) before and (**b**) after ZnS NPs overgrowth. And top view SEM images of CIGS thin film (**c**) before and (**d**) after ZnS NPs overgrowth. Also, SEM images with a tilted view over CIGS thin film (**e**) before and (**f**) after ZnS NPs overgrowth.

Scanning electron microscope (SEM) image (Figure 3) shows a sequence comparison of CIGS thin films before and after ZnS NPs growth. Figure 3b respectively shows photographs of a 2 cm square CIGS thin film with and without the ZnS NPs layer. The thickness of the ZnS NPs layer has been set to several nanometers. The appearance of the CIGS thin film is visually unchanged, showing no severe self-assembly of ZnS NP clusters, and the extremely thin thickness of the ZnS NPs layer. SEM images Figure 3c,d respectively show the CIGS surface morphology before and after ZnS NPs deposition. Figure 3c shows the multiple grain morphology of the CIGS surface. From Figure 3d, a layer of ZnS NPs is observed and appears to be conformally deposited onto CIGS with excellent coverage. Figure 3e,d provide a tilted vision of CIGS morphology change caused by the ZnS NPs layer. Figure 3e shows example CIGS grains with random crystalline facets. Figure 3f shows a thin CIGS film capped by the ZnS NPs layer. The ZnS NPs have only slightly roughened the CIGS surface and preserved most of the intrinsic morphology of the underlying CIGS absorber layer, inferring an extremely thin thickness of the layer. These results confirm homogeneous ZnS NPs layer deposition with very small thickness on a substrate with roughness of tens of nanometers. The thermally decomposed ZnS NPs layer can achieve large-area deposition whilst maintaining a consistently thin film thickness. This is an inherent consequence of ZnS NPs formation. During deposition, the precursor molecules are mostly decomposed at the CIGS surface, where the temperature is highest. ZnS NPs instantly attach to the CIGS surface by Van der Waals interaction, effectively avoiding Van der Waals interaction between particles, and capillary flow of particles, while non-uniform evaporation occurs. These are the two main mechanisms causing nanoparticle aggregation [13,20]. The high coverage of thermally decomposed ZnS NPs on CIGS provide a strong pn junction across the entire 2 cm square rough CIGS surface, without noticeable shunt leakage current, resulting in the high photo-current.

Dark current of the thermally decomposed ZnS NPs device and the CBD ZnS device with the highest efficiency in each category are shown in Figure S2 of Supplementary Information, in which a better coverage of thermally decomposed ZnS NPs is reflected by the lower shunt leakage current obtained from the ZnS NPs device, compared to that of the CBD ZnS device. The higher leakage current from the CBD ZnS device could be caused by the complex mixtures in the CBD ZnS layer, or its inhomogeneous growth on CIGS. Particularly, around the operating voltages of the two CIGS PV devices, the thermally decomposed ZnS NPs device exhibits lower series resistance, showing lower defects concentration [21,22]. The preferable electrical characteristic of ZnS NPs current from the CBD ZnS device could be caused by the complex mixtures in the CBD ZnS layer, or its inhomogeneous growth on CIGS. Particularly, around the operating voltages of the two CIGS PV devices, the thermally decomposed ZnS NPs device exhibits lower series resistance, showing lower defects concentration [21,22]. The preferable electrical characteristic of the ZnS NPs device suggests that the 2 nm single-crystal thermally decomposed ZnS NPs layer effectively protects the CIGS surface from plasma damage during AZO deposition [23,24].

**Figure 4:**
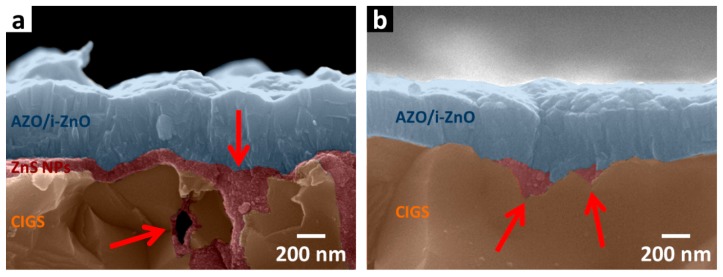
(**a**) Cross-sectional SEM images of CIGS solar cells with (**a**) thick and (**b**) thin layer of ZnS NPs. CIGS. ZnS NPs, and the AZO/i-ZnO window layer have been correspondingly labeled by brown, red and blue shades, while ZnS NPs coated in valley and air voids are particularly indicated by the red arrows.

Cross-sectional SEM images of the thermally decomposed ZnS NPs device have also been examined to investigate the structural quality. Two devices with different thermally decomposed ZnS NP layer thicknesses were prepared. Results are shown in Figure 4a,b where the CIGS layer, thermally decomposed ZnS NPs layer, and the window layer are correspondingly labeled by brown, red and blue shades. In Figure 4a, where SEM image of a CIGS device with the thicker thermally decomposed ZnS NPs layer is shown, a thermally decomposed ZnS NPs layer with thickness around 100 nm can be clearly observed. The inner walls of the valley and air void at the CIGS grain boundary were found to be coated by a thermally decomposed ZnS NPs layer (indicated by the red arrows).

As shown in Figure 4b, the same phenomenon is observed on the thermally decomposed ZnS NPs device with a thinner thermally decomposed ZnS NPs layer on CIGS surface, even though the thermally decomposed ZnS NPs layer here is very thin (with the same thickness as the best thermally decomposed ZnS NPs device) and cannot be observed. This capability to provide inner wall passivation effectively eliminates surface states at CIGS grain boundaries by providing better adhesion of the top AZO layer to the pn junction, which enhances carrier transportation.

**Figure 5:**
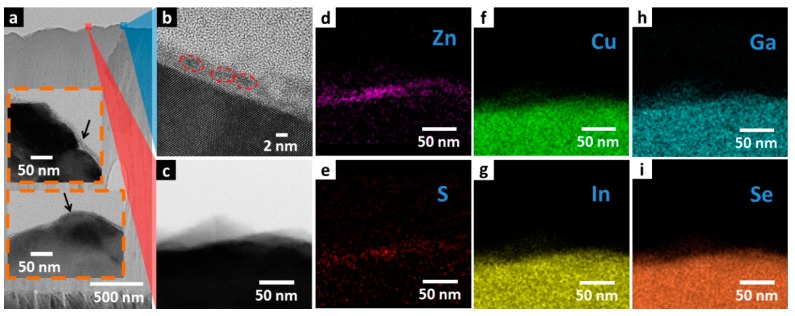
(**a**) TEM overview image of CIGS absorber layer capped by the ZnS NPs layer. Insets show magnified view at ZnS NPs/CIGS interface. (**b**) TEM image of ZnS NPs/CIGS pn junction magnified at individual ZnS NPs (indicated by the blue shade), in which single ZnS NPs are marked by red dashed circles. (**c**) TEM image of where energy dispersive spectrometer (EDS) mapping is conducted, indicated by the red shade, and EDS mapping of (**d**) Zn, (**e**) S, (**f**) Cu, (**g**) In, (**h**) Ga, and (**i**) Se.

Cross-sectional TEM images and energy dispersive spectrometer (EDS) mapping has been performed on thermally decomposed ZnS NPs/CIGS junction material to examine the crystallinity and distribution of thermally decomposed ZnS NPs on CIGS surface and shown in Figure 5. Figure 5a provides a view of thermally decomposed ZnS NPs/CIGS junction material spanning about 1 μm in which the homogeneous thermally decomposed ZnS NPs layer is deposited on CIGS. Insets to Figure 5a show magnified images at the CIGS/ZnS interface, in which the conformal coating of the ZnS layer has been indicated by black arrows. A high-resolution TEM (HRTEM) is shown in Figure 5b providing magnification of the thermally decomposed ZnS NPs layer at the position of the label in Figure 5a by the blue shade. HRTEM image shows that, the thermally decomposed ZnS NPs layer has thickness of 2–3 nm, coinciding with the diameter of single thermally decomposed ZnS NPs observed in Figure 1, suggesting mono-layer deposition of the thermally decomposed ZnS NPs. The atomic pattern of thermally decomposed ZnS NPs has also been shown in HRTEM image. Individual thermally decomposed ZnS NPs with clear boundary have been marked by the red dashed circles. Clear atomic pattern has been observed over the entire thermally decomposed ZnS NPs layer.

EDS mapping was performed at the position indicated by the red shade in Figure 5a, and displayed by the TEM image shown in Figure 5c. Figure 5d,e is EDS map plots of ZnS and S. The two maps show exactly the same spatial distribution concentrated at the CIGS surface, showing that EDS plots of Zn and S were sourced from the same thermally decomposed NPs layer on top confirming its identity. Figure 5f–i shows the EDS map plots of Cu, In, Ga, and Se. The four EDS maps exhibit identical EDS plot distribution, which were collected from the CIGS layer beneath. These results of elemental distribution and TEM analysis show that the single-crystal thermally decomposed ZnS NPs are mostly distributed as a single-layer thin film with thickness down to 2 nm, and has good crystallinity. In contrast, the crystallinity of CBD ZnS has also been investigated, and shown in Figure S3 in Supplementary Information, in which SEM images and TEM images of CBD ZnS/CIGS are shown. The CBD ZnS layer on CIGS was found to be around 20 nm, and is amorphous. Given that the CBD ZnS device has higher shunt leakage current than that of the thermally decomposed ZnS NPs layer, the built-in potential in the CBD ZnS/CIGS junction is smaller than that of the thermally decomposed ZnS NPs junction, even though CBD ZnS is ten times thicker than thermally decomposed ZnS NPs, probably caused by impurity complex and defects.

This difference in crystallinity discriminates device performance of thermally decomposed and CBD ZnS-based devices under high temperature operation. Temperature-dependent J–V measurements (shown in Figure S3 in Supplementary Information) at 150 °C and 250 °C on CBD ZnS devices and thermally decomposed ZnS NPs devices show that CBD ZnS devices deteriorate more severely than thermally decomposed ZnS NPs devices, backing up our deductions on crystallinity from TEM results presented in the previous section.

**Figure 6:**
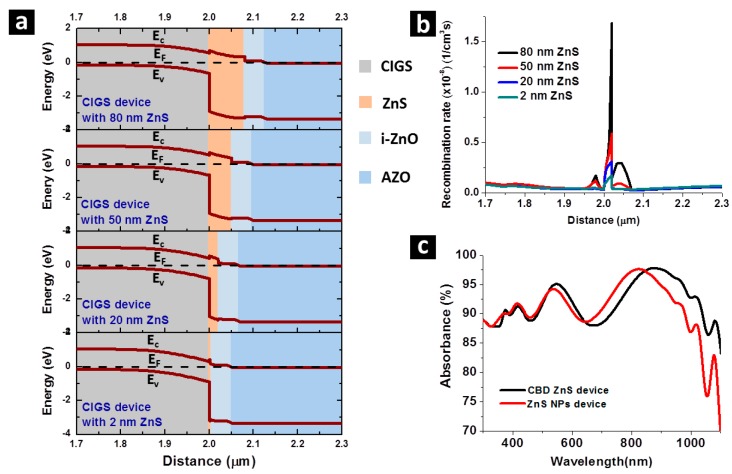
Calculated (**a**) band diagram, and (**b**) carrier recombination rate around CIGS/ZnS interface of CIGS solar cell with 2 nm, 20 nm, 50 nm and 80 nm ZnS buffer layer. (**c**) Absorbance spectra of CIGS solar cells with CBD ZnS buffer layer and ZnS NPs buffer layer.

A thinner n-type buffer layer provided by the thermally decomposed ZnS NPs layer to a CIGS solar cell gives a lower carrier recombination probability at the ZnS/CIGS interface, where a potential barrier is formed for the conduction band [25]. Photon-generated electrons can more easily leap over this potential barrier by thermal excitation if the barrier becomes thinner. This advantage has been verified by numerical calculation using Advanced Physical Models of Semiconductor Devices (APSYS) simulation software and shown in Figure 6. In Figure 6a, band diagram of CIGS solar cells near the thermally decomposed ZnS buffer layer, with a thermally decomposed ZnS buffer layer of 2 nm, 20 nm, 50 nm and 80 nm obtained by simulation are displayed, in which the potential barrier at CIGS/ZnS interface can be seen. The thermally decomposed ZnS layers of 2 nm and 20 nm are constructed in compliance with the thickness of thermally decomposed ZnS in the thermally decomposed ZnS NPs device and the CBD ZnS device, respectively, while 50 nm and 80 nm thermally decomposed ZnS are included as a reference, since these thicknesses of thermally decomposed ZnS in CIGS are also conventionally used. As the thickness of ZnS gets wider, the width of the potential barrier also becomes larger. The carrier recombination rate under Air Mass (AM) 1.5G illumination along the same band diagram range shown in Figure 6a is given in Figure 6b, verifying that, as the potential barrier at ZnS gets larger, carrier recombination rate around ZnS drastically rises simultaneously. Around the ZnS layer, the potential barrier for 20 nm ZnS is 2–3 times higher than that of 2 nm ZnS, providing the higher electrical loss in the CBD ZnS device. With the previous findings showing good crystallinity and coverage of ZnS NPs on CIGS, the mechanism of enhanced current collection observed on ZnS NPs devices has been concluded. Additionally, the absorbance spectra of the ZnS NPs device and the CBD ZnS device have been investigated and shown in Figure 6c, in which no noticeable difference can be observed on the two absorbance spectra, showing that the photo-current enhancement of the ZnS NPs device is purely due to electrical benefits without antireflection effect.

## 3. Materials and Methods

### 3.1. Synthesis of ZnS Nanoparticles Layer

Precursor solution has been prepared, with precursor molecule zinc diethyldithiocarbamate {[(C_2_H_5_)_2_NCS_2_]_2_Zn} dispersed in trioctylphosphine (TOP), using 97% zinc diethyldithiocarbamate (329703 ALDRICH), and 97% trioctylphosphine (718165 ALDRICH) purchased from Sigma Aldrich. Before dispensing the precursor solution, Cu(In,Ga)Se_2_ (CIGS) thin film was pre-heated to 250 °C and stabilized under Ar atmosphere in a non-vacuum chamber. Subsequently, 200 μL of the precursor solution has been dropped onto CIGS surface. The reaction forming homogeneous ZnS nanoparticles (ZnS NPs) thin film takes 5 min. The TOP solvent has been evaporated as the ZnS nanoparticles thin film was ready. Subsequently, acetone was used to wash away residual TOP on the ZnS NPs/CIGS junction sample and accomplish the ZnS NPs layer on CIGS.

### 3.2. Processing of CIGS Solar Cells

Fabrication of the CIGS solar cells started with an 800 nm-thick Mo back-contact prepared by DC-magnetron sputtering on rigid soda lime glass (SLG) with a 2 cm square area. The CIGS absorber layer with thickness of 2 μm was deposited onto Mo-coated SLG substrate by a three-stage co-evaporation process at about 550 °C. Subsequently, a ZnS(O,OH) buffer layer was deposited onto CIGS surface by a 30-min chemical bath deposition (CBD) at 75 °C using a ZnSO_4_/ammonia/thiourea aqueous solution. Optimization of device performance results in the ZnS(O,OH) buffer layer with thickness of 20 nm, forming the CBD ZnS/CIGS junction material. For CIGS solar cells with the ZnS NPs device, the buffer was prepared through the method illustrated in the “Synthesis of ZnS nanoparticles layer” section above. A 50 nm-thick intrinsic ZnO (i-ZnO) and a 250 nm-thick Al-doped Al:ZnO (AZO) stacking layers were subsequently deposited onto the junction materials using RF sputtering. For CIGS solar cells with the CBD-deposited ZnS(O,OH) buffer layer, AZO thin films were deposited under room temperature, while for those with the ZnS NPs buffer layer, AZO thin films were deposited under 150 °C.

### 3.3. Electrical Characterization on CIGS Solar Cells

Current–voltage (J–V) measurements were performed following the procedure described in the international standard CEI IEC 60904-1. All solar cells were characterized by J–V measurements under a simulated Air Mass 1.5 Global (AM 1.5G) illumination with a power of 1000 W/m^2^. The temperature was actively controlled during the measurements and was kept at 25 ± 1 °C. The efficiency and other electrical parameters of CIGS solar cells were obtained by a system consisting of a 1000 W Class A solar simulator (Newport 91192A, Irvine, CA, USA) equipped with a Xenon lamp (Newport 6271A, Irvine, CA, USA), an AM 1.5G filter (Newport 81088A, Irvine, CA, USA) and a current–voltage source (Keithley 2400, Cleveland, OH, USA). The spectrum of the solar simulator was measured with a calibrated spectroradiometer (Soma S-2440). The photon conversion efficiency (PCE) of the devices was measured using a 300 W Xenon (Newport 66984, Irvine, CA, USA) light source and a monochromator (Newport 74112, Irvine, CA, USA). The system was calibrated before each measurement with a calibrated silicon photodetector. The PCE measurements were carried out using a lock-in amplifier (Standard Research System, SR830, Stanford Research Systems, Sunnyvale, CA, USA), an optical chopper unit (SR540, Stanford Research Systems, Sunnyvale, CA, USA) operated at 260 Hz and a 1 Ω resistor in shunt connection.

### 3.4. Absorption Measurements

A commercial UV-Vis-NIR spectrophotometer (Hitachi U4100, Tokyo, Japan) equipped with standard mirror optics and an integrating sphere was used to measure the absorption of the solar cells.

### 3.5. Numerical Simulation

The software used for simulation was Crosslight APSYS (Burnaby, BC, Canada). APSYS is based on 2D finite element analysis and Poisson equation approach, drift-diffusion model to analyze electrons and holes transportation for structure of photovoltaic devices.

## 4. Conclusions

In this paper, we have presented a novel technique to deposit a large-area, single-crystal, homogeneous, thin-film layer of sulfide nanoparticles (such as ZnS, InS, MoS_2_) on a rough surface with controllable thickness down to nanometer scale. This method is accomplished by heating a precursor solution on a substrate (for only 5 min for the ZnS NPs layer studied in this work) with very low waste liquid produced. Self-assembled clusters occurring on dense nanoparticle layers prepared by conventional deposition methods are eliminated. 

By way of practical demonstration in a realistic device, a mono-layer of ZnS NPs was incorporated as the buffer layer of a CIGS solar cell, substituting the conventional layer prepared through chemical bath deposition (CBD). Scanning electron microscopy (SEM), transmission electron microscopy (TEM) and Energy Dispersive Spectrometer (EDS) analysis has revealed the excellent crystallinity of mono-layer ZnS NPs with perfect coverage on the rough surface of a multi-crystalline CIGS thin film. Performance of CIGS solar cells with the new ZnS NPs buffer layer surpasses that of CIGS solar cells with a conventional CBD buffer layer, which has been used by the industry for decades. Through dark current–voltage (J–V) measurement and numerical simulation, it has been verified that the lower electrical loss observed on CIGS solar cells with the ZnS NPs buffer layer is due to the high coverage and high crystallinity of the ZnS NPs layer, and its small 2 nm thickness. 

For the CIGS absorber layers used in this study, the highest efficiency solar cell with the ZnS NPs buffer layer was 11.44%, while devices with the CBD ZnS buffer layer had efficiency around 8%. This corresponds to a 32% relative increase in efficiency. The quality features of the ZnS NPs layer allow optimization of CIGS solar cell by reduction of i-ZnO thickness, which leads to 14.40% best efficiency, and a 65% relative increase in efficiency compared to CIGS solar cells with the CBD ZnS buffer layer. In contrast to CIGS solar cells with the CBD ZnS buffer layer, CIGS solar cells with the ZnS NPs buffer layer are resistant to heat deterioration due to the high quality of the ZnS NPs layer. The thermally decomposed NP layer when used as a buffer layer in CIGS solar cells not only has better performance, but eliminates the problem of waste liquid produced by CBD. Environmental and cost-efficiency demands for mass production can be fulfilled simultaneously not only for CIGS PV devices, but other thin-film solar cell technologies such as CdTe and Cu_2_ZnSnS_4_ (CZTS). We believe that the “breakthrough” nanoparticle layer deposition process presented in this paper may provide alternatives for a wider range of thin-film coating applications.

## Figures and Tables

**Table 1:** Parameters of CIGS solar cells with CBD ZnS buffer layer and ZnS NPs buffer layer.

	Voc (V)	Jsc (mA/cm^2^)	Fill Factor (%)	η (%)
CBD ZnS 1	0.47	28.94	58.25	7.99
CBD ZnS 2	0.46	29.15	58.22	7.85
CBD ZnS 3	0.48	29.60	61.15	8.69
ZnS NPs 1	0.62	30.23	58.85	10.95
ZnS NPs 2	0.60	30.77	53.30	9.90
ZnS NPs 3	0.63	29.97	60.47	11.44

## Supplementary Materials

The following are available online at www.mdpi.com/2079-4991/7/4/78/s1.
