# Supplementary material for: Breakthrough to Non-Vacuum Deposition of Single-Crystal, Ultra-Thin, Homogeneous Nanoparticle Layers: A Better Alternative to Chemical Bath Deposition and Atomic Layer Deposition
Source: Nanomaterials (Basel). 2017 Apr 6;7(4):78. doi: 10.3390/nano7040078 (PMC5408170; doi:10.3390/nano7040078)
Supplement: Supplementary file 1 [file nanomaterials-07-00078-s001.pdf]

Supplementary Information

Non-vacuum deposition of homogeneous, ultra-thin, single-crystal, nanoparticle layer yielding 32% efficiency improvement for $Cu(In,Ga)Se_2$ photovoltaic

[1,2]Yu-Kuang Liao, [1]Yung-Tsung Liu, [3]Dan-Hua Hsieh, [3]Tien-Lin Shen, [4]Ming-Yang Hsieh, [2]An-Jye Tzou, [2]Shih-Chen Chen, [3]Yu-LinTsai, [1]Wei-Sheng Lin, [1]Sheng-Wen Chan, [5]Yen-Ping Shen, [2]Shun-Jen Cheng, [3]Chyong-Hua Chen, [2]Kaung-Hsiung Wu, [5]Hao-Ming Chen, [4]Shou-Yi Kuo, [*,6]Martin D. B. Charlton, [**,1]Tung-Po Hsieh & [***,3]Hao-Chung Kuo

[1]Green Energy & Environment Research Laboratories, Industrial Technology Research Institute, No. 195, Sec. 4, Chung Hsing Road, Chutung, Hsinchu 31040, Taiwan, R.O.C.

[2]Department of Electro-physics and [3]Department of Photonic & Institute of Electro-Optical Engineering, National Chiao Tung University, No. 1001, University Road, Hsinchu 30010, Taiwan, R.O.C.

[4]Department of Electronic Engineering, Chang-Gung University, No. 259, Wen-Hwa 1st Road, Kwei-Shan, Taoyuang 33302, Taiwan, R.O.C.

[5]Department of Chemistry, National Taiwan University, No.1, Sec. 4, Roosevelt Road,

Taipei 10617, R.O.C.

[6]School of Electronics and Computer Science, University of Southampton, SO17 1BJ

Southampton, UK

*mdbc@ecs.soton.ac.uk

** TP@itri.org.tw

***hckuo@faculty.nctu.edu.tw

Comparison of photon conversion efficiency (PCE) spectra

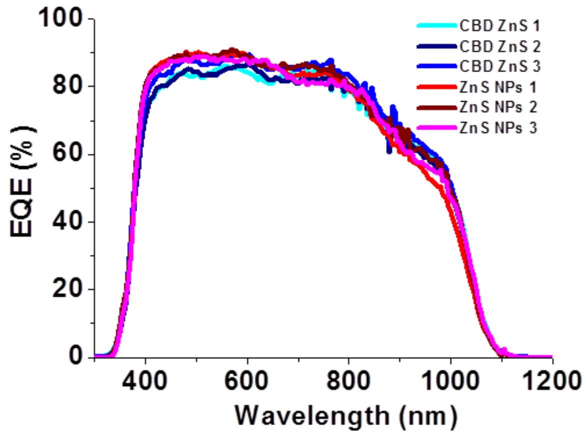

Figure S1 Photon conversion efficiency (PCE) spectra of the three CBD ZnS devices and the three ZnS NPs devices

Figure S1 shows the photon conversion efficiency (PCE) spectra of CBD ZnS devices and ZnS NPs devices. PCE spectra obtained from ZnS NPs device are presented in pink, red and wine red, while curves obtained from CBD ZnS device are presented in blue, light blue and navy blue. Slight PCE enhancements are generally observed on ZnS NPs devices in short wavelength range (400 nm – 650 nm), probably due to that the photocurrent obtained in this range are collected at shallow depth in absorber layer, nearby ZnS/CIGS interface, where ZnS NPs layer with high crystallinity and low defect concentration effects the most. In long wavelengths range (800 nm – 1000 nm), PCE of CBD ZnS devices slightly surpass those of ZnS NPs devices, due to the smaller bandgap energy used for CBD ZnS devices (see main content), which is

capable of harvesting more photons with small photon energies. The intensity of AM1.5G solar spectrum illumination is higher in wavelength range of 400 nm – 650 nm, comparing to longer wavelength range, results in the short circuit current (J_{SC}) enhancement observed on ZnS NPs device.

Dark current of ZnS NPs device and CBD ZnS device

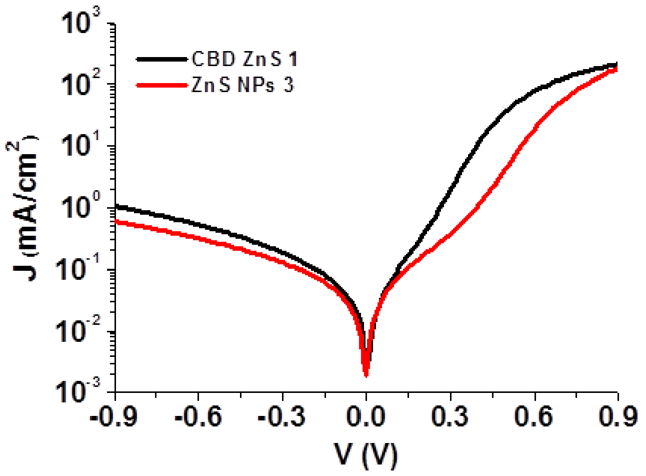

Figure S2 Dark current-voltage (JV) curves of a CBD ZnS device and a ZnS NPs device

Figure S2 shows the dark current-voltage (JV) curves of CBD ZnS device and ZnS NPs device with the highest efficiency separately selected from their categories. The reverse current of CBD ZnS device are higher than that of ZnS NPs device under all reverse bias, showing a higher shunt leakage current. The two devices has different CIGS absorber layer, results in different turn-on voltage (see main content). Around 0.5 V – 0.6 V, where the operating voltage of the two solar cells is located, the slope of JV curve of CBD ZnS device are smaller than that of ZnS NPs device, showing a higher series resistance. These results are due to the higher coverage, and higher crystal quality of ZnS NPs layer, as revealed from the analysis of TEM and SEM shown in main content.

SEM and TEM images of CBD ZnS(O,OH) on CIGS

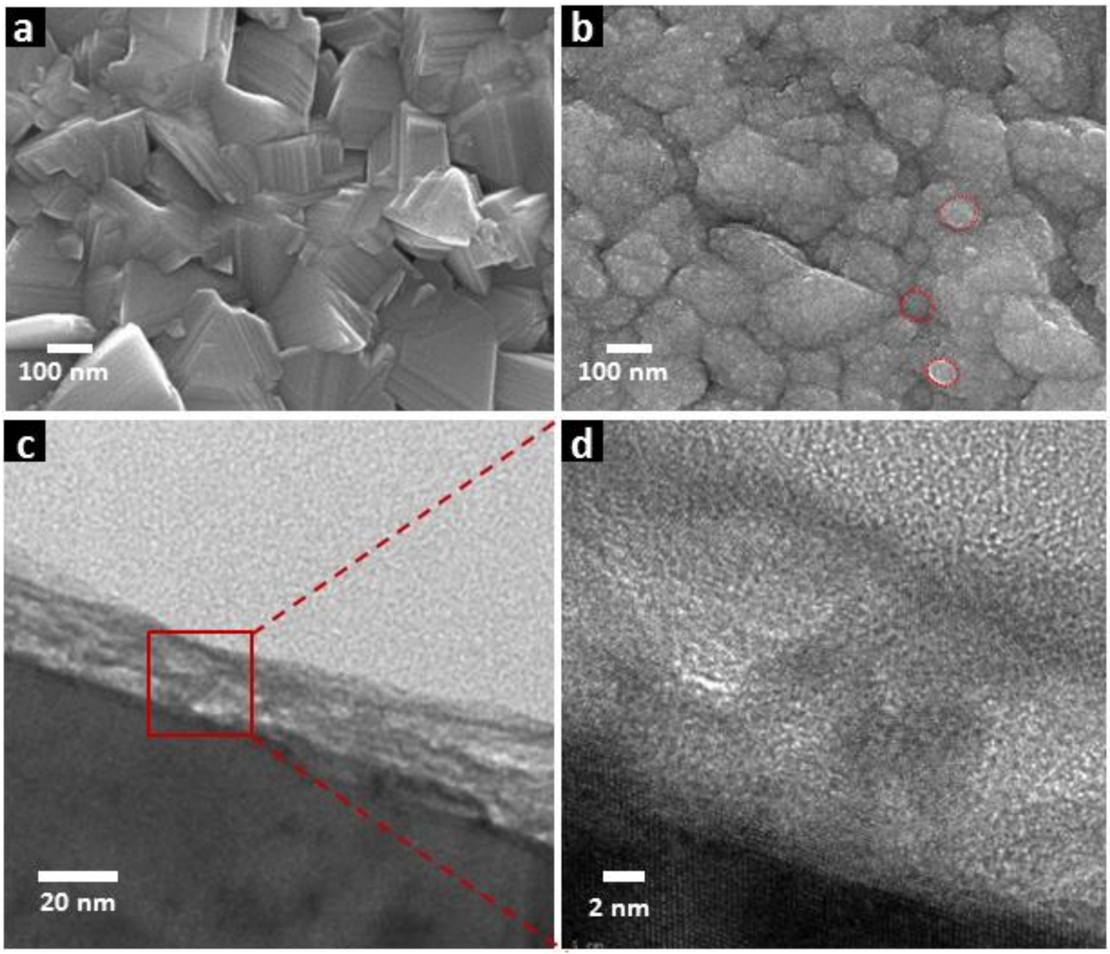

Figure S3 SEM image of CIGS thin film (a) before and (b) after CBD ZnS(O,OH) overgrowth. (c) TEM image of CBD ZnS(O,OH) layer on CIGS, and (d) a magnification image focused at ZnS(O,OH).

The surface morphology of bare CIGS thin film and CIGS thin film capped with CBD ZnS are respectively shown in Figure S3(a) and S3(b). Comparing to that of ZnS NPs layer on CIGS, the surface morphology of CBD ZnS are more quenched due to the larger thickness of CBD ZnS, which is needed to eliminate shunt leakage current.

Many large ZnS particles caused by the more severe aggregation during CBD deposition can been observed on the surface, resulting in non-uniform thickness of CBD ZnS thin film on CIGS, which can brings higher series resistance. Three ZnS aggregated particles have been marked by the red dashed circles for a reference. Figure S3(c) shows a cross sectional TEM image of CBD ZnS on CIGS. A CBD ZnS with thickness of around 20 nm is observed. Figure S3(d) shows a magnification at the CBD ZnS film, in which almost no crystal pattern can be observed, indicating that the CBD ZnS thin film is amorphous. Moreover, from the color contrast exhibited in the TEM image, the CBD ZnS film shows inhomogeneous thickness, partially is even hollow-like, and is probably caused by impurities, and had resulted in a different etching rate during Focus Ion Beam (FIB) etching for TEM sample preparation.

Current-voltage parameters change during annealing

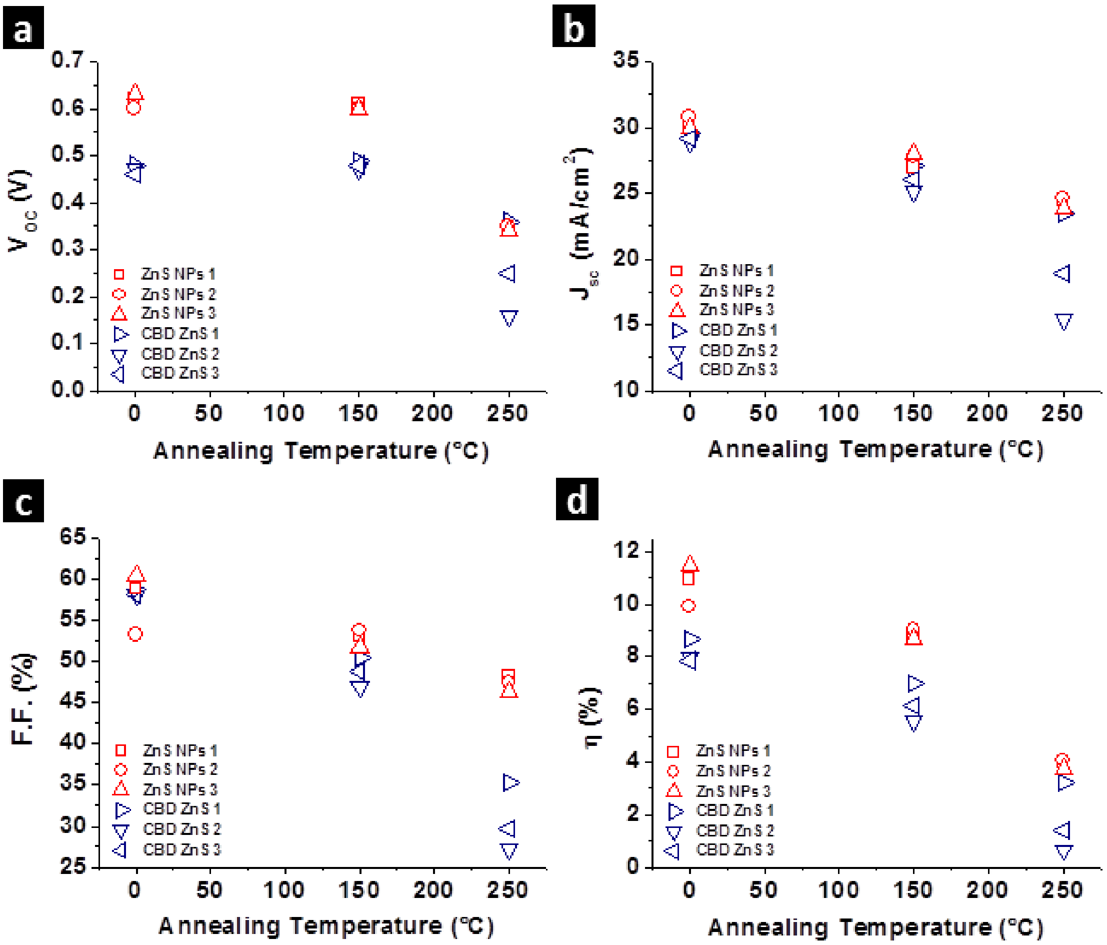

Figure S4 Changes of (a)V_{OC}, (b)J_{SC}, (c) F.F. and (d) efficiency of CIGS solar cells with CBD ZnS buffer layer and ZnS NPs buffer layer during annealing.

The high crystallinity of ZnS NPs buffer layer gives high capacity of heat tolerance, and allows subsequent high growth temperature of AZO deposition, and has also consequently improved the heat tolerance at the PV device performance with ZnS NPs buffer layer. Tests have been made to demonstrate this improvement, for which, the three CBD ZnS devices and the three ZnS NPs devices discussed in Figure 2 in

main content, have been sequentially annealed in an temperature programmable oven at 150˚C and 250˚C. Each annealing lasts for 24 hours and JV measurements have been done after annealing at each temperature. The results are shown in Figure S4, where V_{OC} (Figure S4[a]), J_{SC} (Figure S4[b]), F.F. (Figure S4[c]) and efficiency (Figure S4[d]) are displayed. The initial values of those parameters are presented at zero annealing temperature. High temperature annealing has deteriorated both the two kinds of PV devices, especially at 250˚C. However, as shown in Figure S4, more drastic decrease has been observed on some ZnS CBD devices, revealing their unstable performance under high temperature. This is due to low heat capacity of CBD ZnS buffer layer and AZO layer, both prepared at room temperature. On the contrary, the three ZnS NPs devices have shown consistent change at all parameters, and have performed smaller drop at 250˚C, showing its stability under high temperature environment.
